# Synthesis of Amyloid Images Using a Generative Adversarial Network from 2-Dimensional ^18^F-FDG Images and Evaluation for Clinical Use

**DOI:** 10.2967/jnmt.125.270154

**Published:** 2026-06

**Authors:** Misa Honda, Takahiro Yamada, Shogo Watanabe, Aya Watanabe, Takashi Nagaoka, Mitsutaka Nemoto, Katsuhiro Mikami, Kohei Hanaoka, Hayato Kaida, Hisashi Handa, Kazunari Ishii, Yuichi Kimura

**Affiliations:** 1 Graduate School of Science and Engineering, Kindai University, Osaka, Japan;; 2 Division of Positron Emission Tomography, Institute of Advanced Clinical Medicine, Kindai University Hospital, Osaka, Japan;; 3 National Cerebral and Cardiovascular Center Hospital, Osaka, Japan;; 4 Akita Cerebrospinal and Cardiovascular Center, Akita, Japan;; 5 Faculty of Biology-Oriented Science and Technology, Kindai University, Wakayama, Japan;; 6 Department of Radiology, Faculty of Medicine, Kindai University, Osaka, Japan; and; 7 Faculty of Informatics, Cyber Informatics Research Institute, Kindai University, Osaka, Japan

**Keywords:** Alzheimer disease, generative AI, PET, image processing

## Abstract

The use of amyloid PET to assess patient suitability of disease-modifying drugs for Alzheimer disease is increasing. This study aimed to synthesize amyloid PET images from ^18^F-FDG PET images using a generative artificial intelligence algorithm to reduce unnecessary amyloid PET scans. **Methods:** A 2-dimensional pix2pix algorithm was used. The algorithm was evaluated across 4 domains: image quality, voxel values, contrast between white and gray matter, and diagnostic performance for detecting the presence or absence of β-amyloid (Aβ) deposition. Pairs of ^18^F-FDG PET and amyloid PET images from 55 Aβ-negative and -positive cases were evaluated. A 6-fold cross-validation was conducted. **Results:** Synthetic images were visually consistent, producing plausible negative and positive patterns while preserving continuity in the sagittal plane. Voxel values of the synthetic images showed a significant linear relationship with the real images. The contrast correlated well with the real images, and the differences between the negative and positive cases were significant as well as those in the real images. The performance of the positive or negative 2-class classifier exceeded 85% for the synthetic images. **Conclusion:** The synthetic images successfully captured features of Aβ deposition, and evaluation with a 2-class classifier achieved an acceptable accuracy of 85%. These results suggest that amyloid images can potentially be generated from ^18^F-FDG PET images for use in clinical practice.

## Visual Abstract

**Figure:**
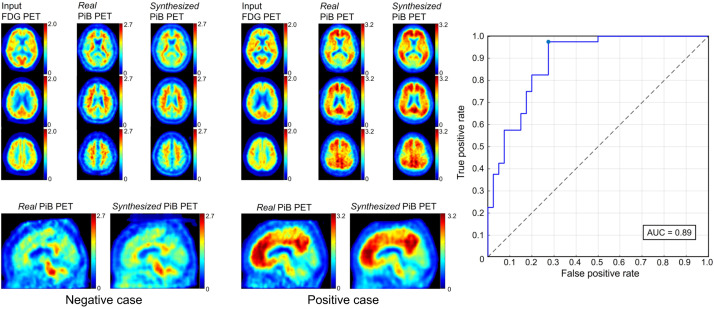

The recent approval of lecanemab, a disease-modifying drug for Alzheimer disease (AD), has brought renewed attention to the treatment of AD, resulting in an increase in the number of amyloid PET scans performed to assess patient suitability for such therapies. If Aβ deposition can be distinguished from images acquired from ^18^F-FDG PET, which shows neuronal activity via glucose metabolism in the brain and is more accessible than amyloid PET, the number of amyloid PET scans could be reduced.

Deep-learning approaches, including generative adversarial networks (GANs), have been explored in previous studies for multimodal synthesis between MRI and PET (1,2) and for applications involving various amyloid tracers (3–5). The training of artificial intelligence–based algorithms, such as GANs, requires thousands of images, which poses a challenge for clinical applications (6). In previous studies, GAN training was performed in 3 dimensions, yielding only 1 training sample per case. In contrast, training a GAN on 2-dimensional (2D) slice-by-slice units generates approximately 50 slices per case, enabling effective learning with a limited number of clinical cases. Although thousands of images are available from public databases, such as the Alzheimer’s Disease Neuroimaging Initiative, their use is limited to research. The use of 2D images allows artificial intelligence–based algorithms to be trained with fewer clinical cases, which is particularly helpful given the difficulty of recruiting large numbers of patients in clinical trials (7).

Amyloid PET visualizes the deposition of β-amyloid (Aβ), which causes AD. Previous studies, such as those by Hu et al. (2) and Choi et al. (4), have used evaluation metrics (mean squared error, structural similarity index measure, and peak signal-to-noise ratio) for the quantitative evaluation of synthetic images that represent the quality of the entire image. Aβ positivity is determined by examining the region where Aβ deposition occurs, such as the contrast between white and gray matter in the posterior cingulate gyrus and frontal lobes, rather than by assessing the entire brain image. Therefore, image evaluation should focus on the specific regions used to detect Aβ deposition instead of the whole brain.

In this study, we synthesized amyloid PET images from ^18^F-FDG PET images using a 2D GAN approach. We quantitatively evaluated synthetic amyloid PET images and investigated their ability to distinguish the presence or absence of Aβ deposition.

## MATERIALS AND METHODS

### Dataset

We analyzed 110 pairs of ^18^F-FDG PET and ^11^C-labeled Pittsburgh compound B (^11^C-PiB) PET images acquired from 55 Aβ-positive and 55 Aβ-negative patients. These cases were acquired from studies conducted between 2011 and 2021 (8–12). This study was performed in accordance with the Declaration of Helsinki. This human study was approved by the Ethics Committee of the Faculty of Medicine (R03-077) and Faculty of Informatics (2023-001) at Kindai University. The use of existing anonymized information and opt-out methods waived the need to obtain written informed consent from the clinical research review committee.

We used 2 PET scanning machines: ECAT Accel (Siemens Medical Solutions) and Discovery PET/CT 710 (GE HealthCare). These PET scanners are qualified by Japanese Society of Nuclear Medicine (13) for Discovery PET/CT 710 or J-ADNI2 for ECAT Accel (UMIN000010991). For the ^18^F-FDG PET examination, all patients fasted for at least 4 h before receiving the ^18^F-FDG injection, and their blood glucose levels were ensured to be normoglycemic (<150 mg/dL). We administered a single intravenous injection of 185 MBq of ^18^F-FDG and instructed the subjects to lie quietly on a couch with their eyes open in a dimly lit room for 30 min with minimal sensory stimulation. Patients were then imaged using a 30-min emission scan. For the amyloid PET scan, patients received an intravenous injection of ^11^C-PiB (mean dose, 555 ± 185 MBq), and a 20-min emission scan was performed 50 min after injection.

We normalized the ^18^F-FDG PET images by their whole brain values and the ^11^C-PiB PET images by their cerebellum gray matter that exhibited Aβ deposition (10). Coregistration accuracy is important, as the algorithm we applied uses paired images for training. We coregistered the 2 images by the registration of ^18^F-FDG PET and ^11^C-PiB PET images to the individual MR images, respectively, using SPM12, a well-established and generally reliable coregistration method (14). To reduce the influence of individual differences in brain shape, all images were anatomically standardized using SPM12.

### Generative Model

**FIGURE 1.**
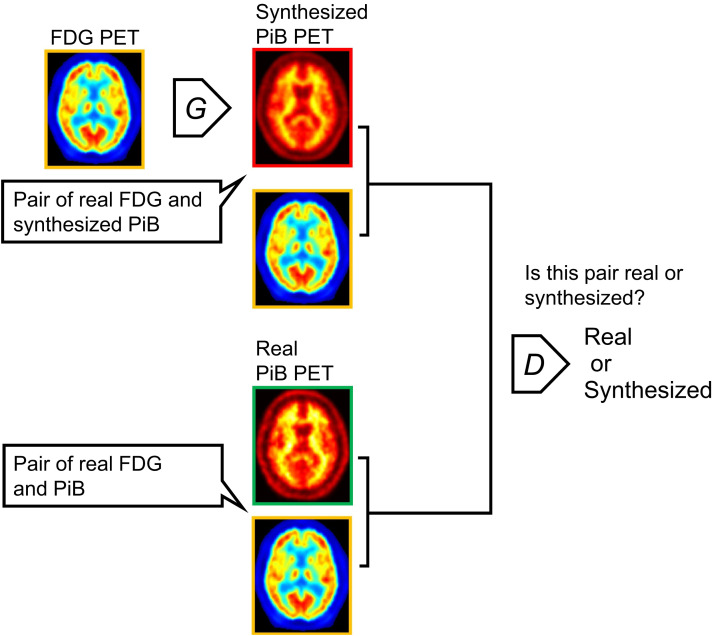
Block diagram of pix2pix. Input was ^18^F-FDG PET image, and output was ^11^C-PiB PET image. D = discriminator; G = generator.

We used pix2pix, a well-established image-synthesizing algorithm based on GAN (15). Figure 1 shows a schematic of the pix2pix network structure. The discriminator, PatchGAN, classified the input image as real or synthetic. The generator, which has a U-Net structure, produced images in which the discriminator cannot distinguish between real and synthetic images. Details of the algorithm are available in Supplemental Figure 1 at http://tech.snmjournals.org.

We implemented the pix2pix algorithm using MATLAB R2023a and the GPU of NVIDIA Quadro RTX8000. We obtained the code of pix2pix from https://jp.mathworks.com/matlabcentral/fileexchange/74779-pix2pix. The pix2pix network was trained using 130 epochs and a batch size of 128. The Adaptive Moment Estimation optimizer was configured with a learning rate of 0.0002, β1 of 0.5, and β2 of 0.999.

A 6-fold cross-validation was performed. Thirty of the total 110 cases were used to train the classifier for Aβ-negative and -positive status; the remaining 80 cases were used to train and validate the pix2pix network through the 6-fold cross-validation process. For both classifier and network training, equal numbers of positive and negative cases were ensured, and an even number of cases (66 or 68) was allocated for network training within each fold. We used 2D slices for training and synthesis to obtain sufficient training data. In 2D, about 50 slices cover the brain region; therefore, more than 3,000 images could be applied to train pix2pix. We resliced the images used for training to 128 × 128 × 60 pixels (the size of the images used in pix2pix). We then created images in axial orientation for training in 2D. It is possible to train a GAN to synthesize PET images in 2D, producing results comparable to those obtained with 3D training (16). We ensured that minibatches for training the pix2pix model were balanced between slices from positive and negative cases. The model was trained using paired images (^18^F-FDG PET and ^11^C-PiB PET) acquired on the same scanner. Accordingly, any differences between scanners were inherently incorporated into the training process.

### Performance Evaluation

**FIGURE 2.**
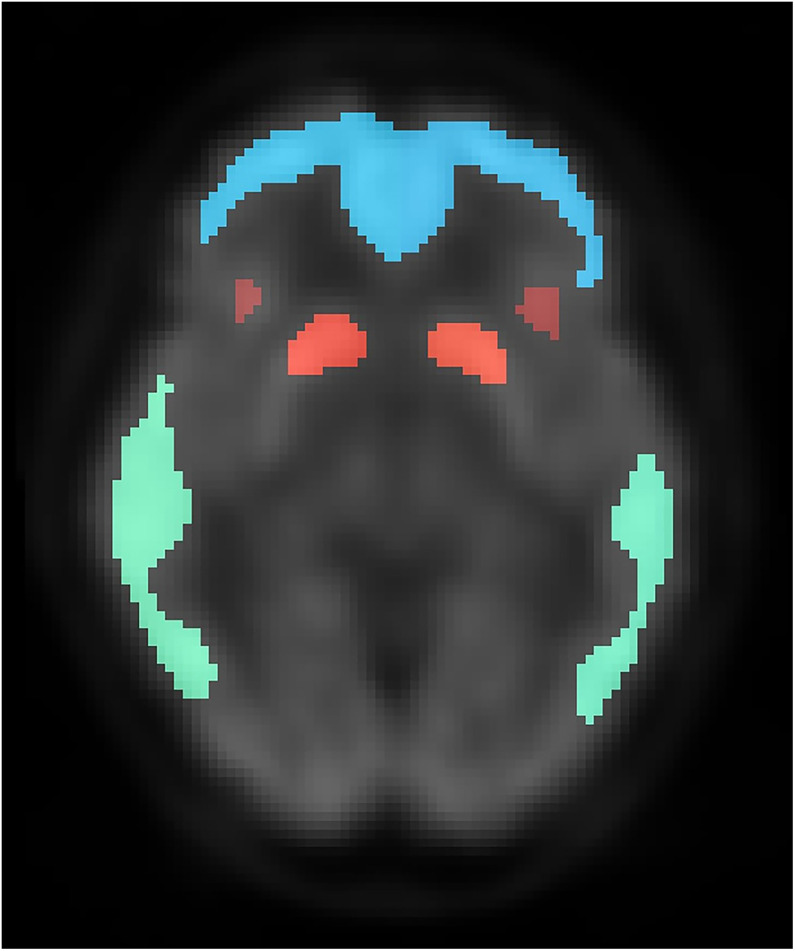
ROI used for quantitative evaluation of pixel values in synthetic image. ROI is shown over ^18^F-FDG PET image. Each area is color-coded. Centiloid ROI was divided into 10 groups bilaterally: frontal/insular gyrus (cyan and red), temporal lobe (green), parietal lobe, posterior cingulate gyrus/precuneus, and striatum (orange).

The algorithm was evaluated across 4 domains: image quality, voxel values, contrast between white and gray matter, and diagnostic performance for detecting the presence or absence of Aβ deposition. Centiloid ROI (17) was used to evaluate voxel values and diagnostic performance because it was proposed for the harmonization of amyloid imaging among multiple PET sites and radiopharmaceuticals and because the ROI was placed onto regions that are relevant for the diagnosis of AD. Figure 2 shows an example of Centiloid ROI used in this study. To assess whether the voxel values in the real and synthetic images were locally consistent, we divided Centiloid ROI into 10 groups bilaterally: frontal/insular gyrus, temporal lobe, parietal lobe, posterior cingulate gyrus/precuneus, and striatum.

**FIGURE 3.**
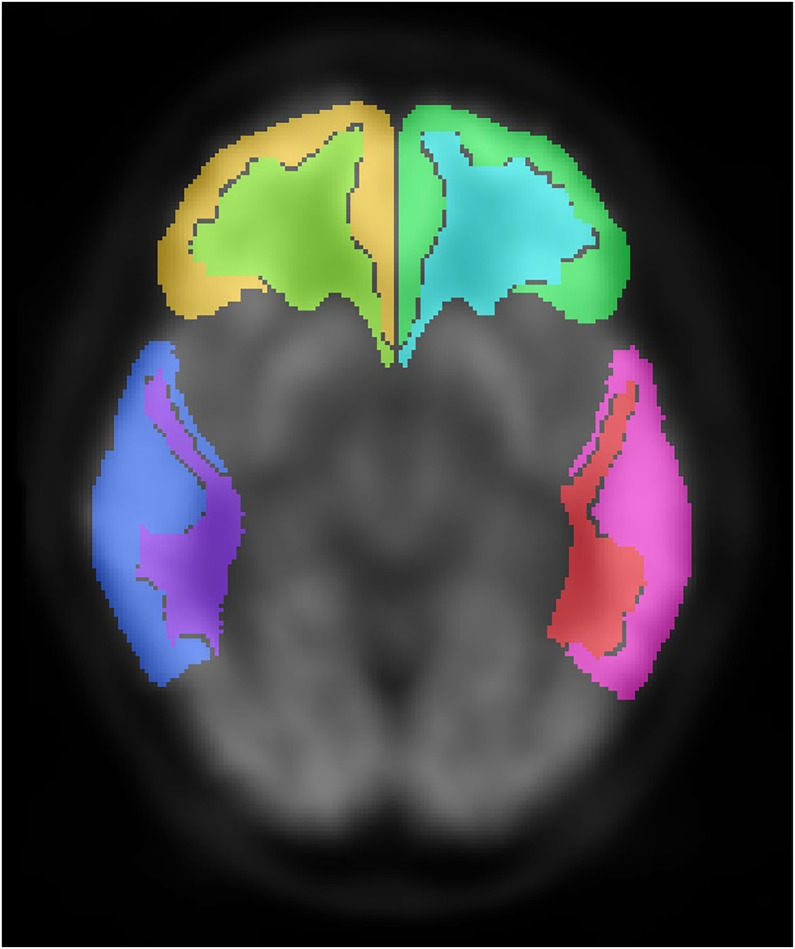
ROI used to assess contrast between white and gray matter. ROI is shown over ^18^F-FDG PET image. Each area is color-coded. ROIs were placed in 4 regions relevant to AD: frontal lobe (yellow, light green, cyan, and dark green), temporal lobe (blue, purple, pink, and orange), parietal lobe, and posterior cingulate gyrus/precuneus. Except for posterior cingulate gyrus and precuneus, ROIs were based on Hammers ROI. Gray matter ROIs in posterior cingulate gyrus and precuneus were created using Automated Anatomic Atlas 3 template, while white matter ROIs were created manually.

Contrast between white and gray matter is a useful assessment method, especially for diagnosis. In Aβ-negative cases, Aβ deposition is greater in white matter than in gray matter, and the deposition in gray matter increases in Aβ-positive cases. We quantified the characteristics of such Aβ deposition between white and gray matter and calculated the contrast as follows:

$$\text{contrast}=\frac{\mu_{W}-\mu_{G}}{\sigma_{W}+\sigma_{G}},$$ Eq. 1

where $\mu_{W}$ and $\mu_{G}$ are the mean values of the white and gray matter, respectively, and $\sigma_{W}$ and $\sigma_{G}$ are the SD of the white and gray matter, respectively. This formula quantifies the relative difference between white and gray matter with respect to their variations. One-sided Wilcoxon rank sum test was used to determine whether there was a significant difference between the contrast values of Aβ-positive and -negative cases in both real and synthetic images. To evaluate the contrast between white and gray matter in the areas relevant to AD, Hammers ROI (18) was used instead of Centiloid ROI, as Centiloid ROI is defined only in gray matter, whereas Hammers ROI covers both white and gray matter. We created a mask for white matter regions from the template originally contained in the template of SPM12. For the posterior cingulate gyrus and precuneus, gray matter regions were derived from the Automated Anatomic Atlas 3 template (19) and white matter regions were manually defined, as this template delineated these regions more clearly than did Hammers ROI. The ROIs used are shown in Figure 3.

We used kernel logistic regression, with a gaussian kernel for quantitative evaluation, to classify Aβ deposition as positive or negative. The features provided to the classifier were mean voxel values acquired from Centiloid ROI. The regressor was trained exclusively on real ^11^C-PiB PET images from 30 cases that were not used for pix2pix training. We first verified the generalization performance of the kernel logistic regression classifier. A 5-fold cross-validation was performed on the data used to train the classifier, resulting in a correct response rate of 97.6%, which was considered acceptable. We then created an exact classifier using all 30 cases to evaluate the discriminatory performance of the synthetic ^11^C-PiB PET images. Image quality metrics, including mean squared error, peak sign-to-noise ratio, and structural similarity index measure, are provided in Supplemental Table 1.

## RESULTS

**FIGURE 4.**
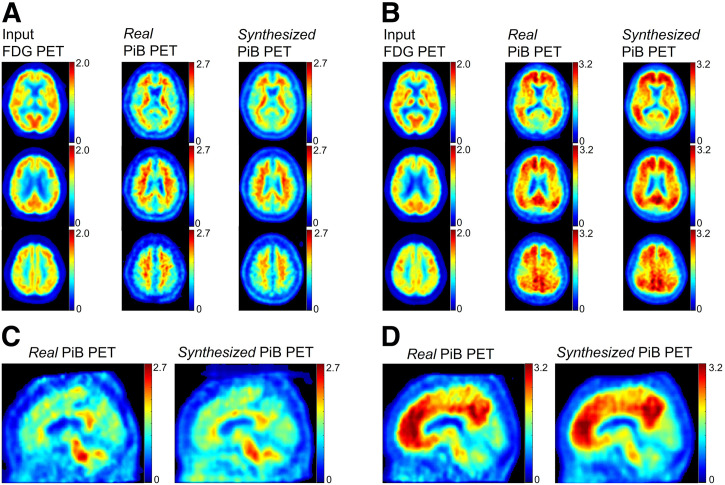
Representative synthetic ^11^C-PiB PET images. Panels A and C are negative for Aβ deposition, whereas panels B and D illustrate Aβ deposition. Panels C and D are cross sections in sagittal plane reconstructed from synthetic axial slices.

Representative synthetic images are shown in Figure 4. ^11^C-PiB accumulates in the white matter as a result of nondisplaceable binding of ^11^C-PiB. In cases with positive Aβ deposition, the accumulation of ^11^C-PiB is observed in both white and gray matter, which is visually indistinguishable. Synthetic ^11^C-PiB PET images in Aβ-negative patients showed a typical negative pattern, with ^11^C-PiB accumulating mainly in the white matter, whereas synthetic ^11^C-PiB PET images of positive cases show a typical positive finding, with accumulation in both the gray matter and white matter. Figures 4C and 4D are reconstructed images to stack synthetic axial images and indicate continuity along the body axis.

**FIGURE 5.**
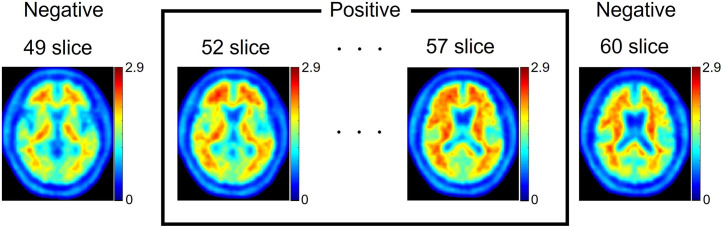
Example of failed image synthesis (negative for Aβ deposition). Negative images were synthesized from most slices. However, slices 52–57 exhibit appearance positive for Aβ deposition.

In a few cases, only some consecutive slices were synthesized with a diagnosis inconsistent with the actual diagnosis. A representative example is shown in Figure 5.

For the quantitative evaluation of voxel values, a significant linear relationship was found (*P* < 0.05) between the real and synthetic images for all folds. For all folds, the slope ranged from 0.36 to 0.60, the *y*-intercept ranged from 0.54 to 1.00, and the mean correlation coefficient was 0.53.

**FIGURE 6.**
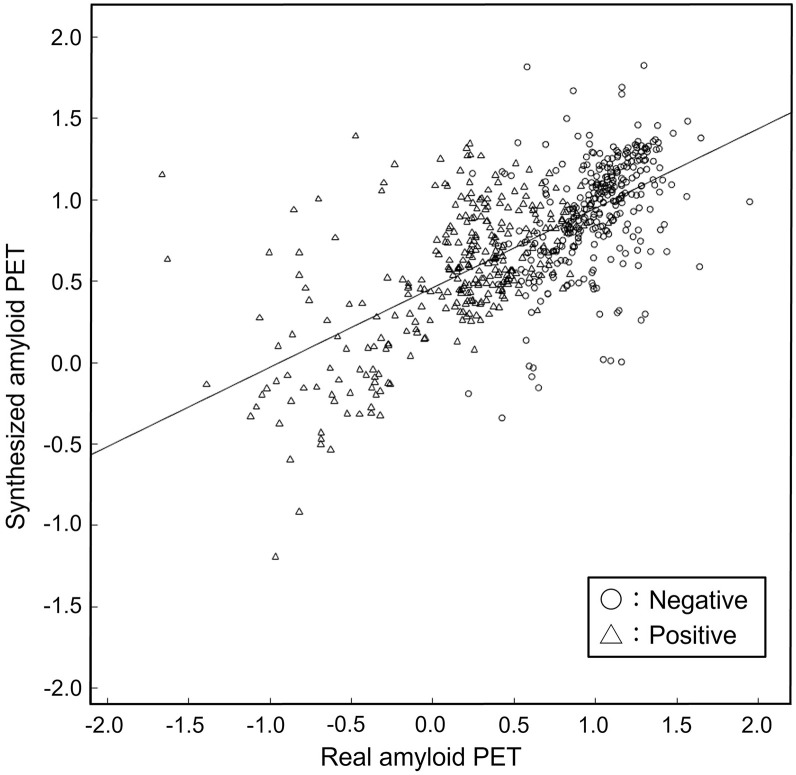
Relationship of contrast between white matter and gray matter to real and synthesized images. Each plot point represents contrast value for each ROI. Regression line is $y=0.49x+0.46\;(r^{2}=0.45)$. 95% CIs for slope and *y*-intercept are 0.45–0.53 and 0.43–0.49, respectively.

Figure 6 shows the contrast between white and gray matter in the real and synthetic images. This is the pooled result of all ROIs and folds. The coefficients of the regression lines for each fold are shown in Table 1. The regression lines at all folds were significant. We found significant differences between positive and negative cases in the synthetic images and in the real images.

**TABLE 1.** Linear Regression of Contrast of White and Gray Matter Between Real and Synthetic Images in Each Fold

Fold no.	Slope	*y*-intercept	*r* ^2^
1	0.51 (0.42–0.59)	0.44 (0.37–0.52)	0.56
2	0.51 (0.40–0.62)	0.41 (0.32–0.50)	0.43
3	0.26 (0.15–0.37)	0.74 (0.65–0.83)	0.19
4	0.55 (0.44–0.65)	0.48 (0.40–0.56)	0.51
5	0.59 (0.49–0.68)	0.22 (0.15–0.30)	0.61
6	0.57 (0.49–0.65)	0.42 (0.36–0.49)	0.65

Values in parentheses are 95% CI.

**FIGURE 7.**
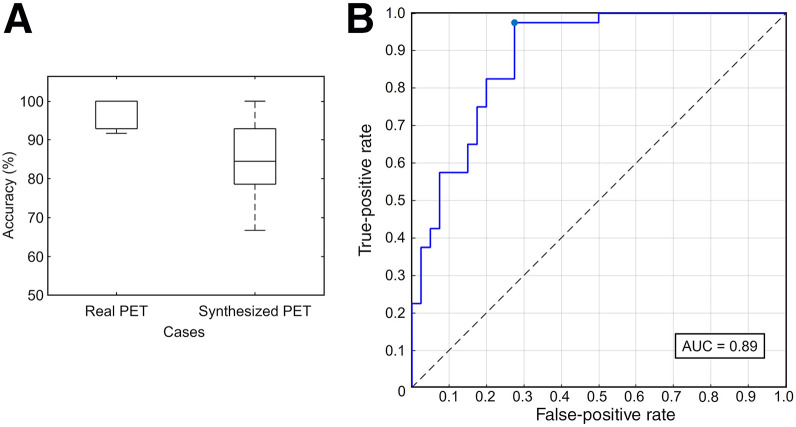
Results for 2-class classifiers that judge given amyloid image as negative or positive. Panel A shows accuracy for each fold. Panel B shows receiver-operating-characteristic curve (AUC) for synthetic image.

Figure 7 illustrates the results for 2-class classifiers: the percentage of correct responses for each fold in the real and synthetic images and the receiver-operating-characteristic curve for synthetic images. Classification accuracy was higher real images than for synthetic images; however, accuracy for synthetic images remained acceptable at approximately 85% (68/80). For synthetic images, sensitivity was 73% (29/40) and specificity was 98% (39/40), with an area under the receiver-operating-characteristic curve of 0.89. These results indicate that it is possible to determine the presence or absence of Aβ deposition using synthetic images.

## DISCUSSION

This study investigated the possibility of replacing widely used amyloid PET scans with ^18^F-FDG PET images.

We used pix2pix to synthesize amyloid PET images from ^18^F-FDG PET images. Although pix2pix is expected to perform well as an image synthesizer, it requires many training images. In medical applications, the maintenance of large datasets spanning diverse subject backgrounds—such as disease severity, sex, age, and race—is often impractical. If we train pix2pix slice-by-slice, an adequate number of images can be maintained because about 50 slices cover the brain region. However, continuity along a 3-dimensional direction should be investigated in synthetic images. In this study, we performed a comparative evaluation of real and synthetic images, focusing on clinically relevant criteria for detecting Aβ deposition. As a result, the accuracy of the 2-class classifier was 85%, which was considered acceptable.

We evaluated the synthetic images across 4 domains: image quality, voxel values, contrast between white and gray matter, and diagnostic performance for detecting the presence or absence of Aβ deposition. Our algorithm synthesized visually reasonable negative and positive images. Surprisingly, continuity in the sagittal plane was well-preserved (Fig. 4), suggesting that results comparable to those obtained with 3-dimensional training can also be achieved with 2D training. The voxel values and contrast between white and gray matter in the synthetic images demonstrated linear correlation with those in the real images (Fig. 6). Regarding the contrast, the significant differences between positive and negative cases in the synthetic images were found as well as in the real images. 　

When evaluating practical use, the diagnostic performance of Aβ deposition reached an acceptable level (Fig. 7). The classifier can be regarded as an artificial neuroradiologist, and our synthetic amyloid PET images exhibited a diagnostic performance similar to that of real images; the performance was lower than that of real cases but still acceptable at more than 85%. Furthermore, we demonstrated that it is possible to synthesize images that retain Aβ-positive and -negative features, allowing accurate assessment of Aβ accumulation (i.e., positive or negative).

Our algorithm has 2 limitations. First, the structural similarity index measures of our images were smaller than those used in previous works (0.54–0.74 vs. 0.75–0.91) (5,6); however, the synthetic amyloid images were clinically acceptable (accuracy of 85%) (Fig. 7). Second, in certain cases, a few consecutive slices were synthesized as a diagnosis opposite to the actual diagnosis (Fig. 5). Although voxelwise quantitative evaluation showed a correlation between real and synthetic images, the result was suboptimal. Similarly, in the contrast-based quantitative evaluation, the correlation between the real and synthetic images was poor for only 1-fold (Table 1). These discrepancies are likely a result of the lack of information along the body axis, as training and synthesis were performed in 2D. To address this, we intend to train pix2pix using additional slice information along the body axis in future studies. This can be achieved by inputting 3 or more consecutive slices during pix2pix training or by adding slices in the sagittal or coronal plane.

Finally, several steps are needed before applying our algorithm in a clinical setting. Larger and more diverse datasets—including a broader range of ages, sexes, and scanner types—will be necessary to verify the clinical generalizability of the image synthesis algorithm for AD diagnosis. Regarding PET scanners, our current dataset does not include images from the latest PET systems. Continuous updates of our model with data from newer scanners will be important, as these systems offer higher spatial resolution and advanced reconstruction algorithms.

## CONCLUSION

This study demonstrates the feasibility of synthesizing amyloid PET images from ^18^F-FDG PET images using a 2D approach. The synthetic images successfully captured features of Aβ deposition, and evaluation with a 2-class classifier achieved an acceptable accuracy of 85%. These results suggest that amyloid images can potentially be generated from ^18^F-FDG PET scans for use in clinical practice.

## DISCLOSURE

This study was conducted under the Research Cluster of Health-Longevity-Development Research Core in Kindai University (4-22) and was supported by JST Support for Pioneering Research Initiated by the Next Generation (SPRING), Japan (grant JPMJSP2182). No other potential conflict of interest relevant to this article was reported.

## Notes

KEY POINTS **QUESTION:** Is it possible to synthesize amyloid images from ^18^F-FDG images using GAN in 2D for clinical applications? **PERTINENT FINDINGS:** Synthetic images successfully captured features of Aβ deposition, and evaluation with a 2-class classifier achieved an acceptable accuracy of 85%. These results suggest that amyloid images can potentially be generated from ^18^F-FDG PET scans for use in clinical practice. **IMPLICATIONS FOR PATIENT CARE:** Amyloid images generated from ^18^F-FDG PET scans could reduce the number of amyloid PET scans performed, improve patient access to accurate amyloid testing, and enhance identification of individuals suitable for AD therapies.
